# Bidirectional interplay of HSF1 degradation and UPR activation promotes tau hyperphosphorylation

**DOI:** 10.1371/journal.pgen.1006849

**Published:** 2017-07-05

**Authors:** Eunhee Kim, Kazuko Sakata, Francesca-Fang Liao

**Affiliations:** Departments of Pharmacology and Department of Anatomy and Neurobiology, TSRB 218A, University of Tennessee Health Science Center, Memphis, Tennessee, United States of America; University of California, San Francisco, UNITED STATES

## Abstract

The unfolded protein response (UPR) in the endoplasmic reticulum (ER) and the cytoplasmic heat stress response are two major stress response systems necessary for maintaining proteostasis for cellular health. Failure of either of these systems, such as in sustained UPR activation or in insufficient heat shock response activation, can lead to the development of neurodegeneration. Alleviation of ER stress and enhancement of heat shock response through heat shock factor 1 (HSF1) activation have previously been considered as attractive potential therapeutic targets for Alzheimer’s disease (AD)—a prevalent and devastating tauopathy. Understanding the interplay of the two aforementioned systems and their cooperative role in AD remain elusive. Here we report studies in human brain and tau pathogenic mouse models (rTg4510, PS19, and rTg21221), identifying HSF1 degradation and UPR activation as precursors of aberrant tau pathogenesis. We demonstrate that chemical ER stress inducers caused autophagy-lysosomal HSF1 degradation, resulting in tau hyperphosphorylation in rat primary neurons. In addition, permanent HSF1 loss reversely causes chronic UPR activation, leading to aberrant tau phosphorylation and aggregation in the hippocampus of aged HSF1 heterozygous knock-out mice. The deleterious interplay of UPR activation and HSF1 loss is exacerbated in N2a cells stably overexpressing a pro-aggregation mutant Tau_RD_ ΔK280 (N2a-Tau_RD_ ΔK280). We provide evidence of how these two stress response systems are intrinsically interweaved by showing that the gene encoding C/EBP-homologous protein (CHOP) activation in the UPR apoptotic pathway facilitates HSF1 degradation, which likely further contributes to prolonged UPR via ER chaperone HSP70 a5 (BiP/GRP78) suppression. Upregulating HSF1 relieves the tau toxicity in N2a-Tau_RD_ ΔK280 by reducing CHOP and increasing HSP70 a5 (BiP/GRP78). Our work reveals how the bidirectional crosstalk between the two stress response systems promotes early tau pathology and identifies HSF1 being one likely key player in both systems.

## Author summary

Tauopathy including Alzheimer’s disease (AD) is characterized by a build-up of tau aggregates in the brain, highly associated with failure of cellular protein homeostasis. Proteostasis can be achieved by protein quality control system to cope with numerous stresses such as proteotoxic stress from misfolded proteins. This cellular protective system includes heat shock response regulated by heat shock factor 1 (HSF1) activation and unfolded protein response in ER. Despite the importance of stress response in maintaining proteostasis, their role in neurodegenerative diseases like tauopathy is not clearly understood. The current study reports how the interplay between the two stress response systems, unfolded protein response and HSF1 promotes early tau pathology and identifies HSF1 protein degradation being one likely key player in both human AD and tau transgenic mouse AD models. We identify aging-associated AD-like neuropathological changes in the hippocampus of HSF1 heterozygous knock-out mice. We speculate that that HSF1 loss may constitute a mechanistic connection between ER stress and tau hyperphosphorylation in tau pathology. This study demonstrates the potential therapeutic significance of stabilizing HSF1 protein in treating AD.

## Introduction

Neurofibrillary tangles (NFTs) of phosphorylated tau aggregates and senile plaques of amyloid beta (Aβ) are the pathological hallmarks of Alzheimer’s disease (AD) patients. Aβ toxicity has been known to contribute to synaptic loss and cognitive impairment, the mechanism of which appears to be tau-dependent [1, 2, 3]. However, NFTs, not amyloid plaques, have been identified to correlate best with the severity of dementia [4, 5]. NFTs exist primarily inside the cell, and has been associated with cellular stress responses [6, 7, 8]. Such response systems include unfolded protein response (UPR) initiated in the endoplasmic reticulum (ER) and cytoplasmic heat shock response initiated by heat shock factor 1 (HSF1) activation. Both pathways involve transcriptional activation of the stress-response genes. A key feature of heat shock response is to induce a set of molecular chaperone proteins such as Hsp70 whose function is to correct protein folding in response to numerous cellular stresses. Likewise, UPR is initially triggered as an adaptive response to disturbances in ER homeostasis. In this sense, HSP70 a5 (BiP/GRP78), a major ER chaperone Hsp70, has been found to attenuate ER stress by activating sensors of transmembrane ER stress, such as protein kinase RNA-like endoplasmic reticulum kinase (PERK), via direct binding [9]. However, sustained chronic UPR activation as a result of unresolved ER stress can eventually trigger cell death by inducing pro-apoptotic proteins such as C/EBP homologous protein (CHOP) primarily through the PERK/eIF2α/ATF4 pathway [10, 11, 12]. A close correlation between ER stress markers and NFTs has been consistently reported in human tauopathies such as AD and frontotemporal dementia [7, 13, 14]. Ho *et al*., 2012 has shown that ER stress can cause tau hyperphosphorylation in primary cultured neurons [15]. However, it is unknown whether and how ER stress causes tau phosphorylation, and vice versa. While these two stress systems (i.e. heat shock response, UPR) have been originally considered to be individually triggered by distinct stressors, recent studies have begun to highlight the importance of heat shock responses in relieving ER stress in non-neuronal cells [11, 16, 17, 18]. Nevertheless, how ER stress affects HSF1-mediated stress response is poorly understood, particularly in the contexts of neurons and tauopathy.

We recently identified aberrant HSF1 degradation via ubiquitin proteasome system as an important mechanism underlying synucleinopathy [19]. Synucleinopathy has been suggested to be pathogenetically linked with tauopathy as reflected by their frequent co-occurrence in neurodegenerative diseases and the synergistic interaction of tau and synuclein [20, 21, 22]. Tauopathy is characterized by a build-up of tau aggregates, and thus the notion that HSF1 could also be degraded by tau aggregation stands as an intriguing possibility. In an attempt to understand human AD tau pathology, experimental murine tauopathy has been generated by introducing mutations in the human tau gene causing frontotemporal dementia and parkinsonism linked to chromosome 17 (FTDP-17) such as P301L, P301S, and ΔK280 [23, 24, 25]. Presence of UPR activation in the tau transgenic mouse models has not been clearly defined. Spatara *et al*., 2010 reported that PS19 mice harboring human tau P301S variant did not show any signs of UPR activation, whereas Abisambra *et al*., *2013* provided some evidence of UPR activation in rTg4510 mice overexpressing human tau carrying the P301L mutation [3, 26]. There is a study showing that Tau ΔK280 mutation aggregates faster than any other single missense mutation [27]. Tau ΔK280 mutation has never been discussed in terms of its involvement of UPR activation. Here, we examined the impact of ER stress-induced UPR activation on HSF1 protein and vice versa in promoting aberrant tau pathology in AD. We investigated how UPR effector proteins such as CHOP and HSP70 a5 (BiP/GRP78) regulate HSF1 to potentiate a vicious cycle active in the cellular tauopathy model (N2a-Tau_RD_ΔK280). Our work highlights that HSF1 loss may constitute a mechanistic connection between ER stress and tau hyperphosphorylation in tau pathogenesis.

## Results

### Loss of HSF1 protein is an early event that precedes NFTs formation in AD, which is further exacerbated in the presence of PERK (or UPR) activation in the brains of mouse and human tauopathy

**Fig 1:**
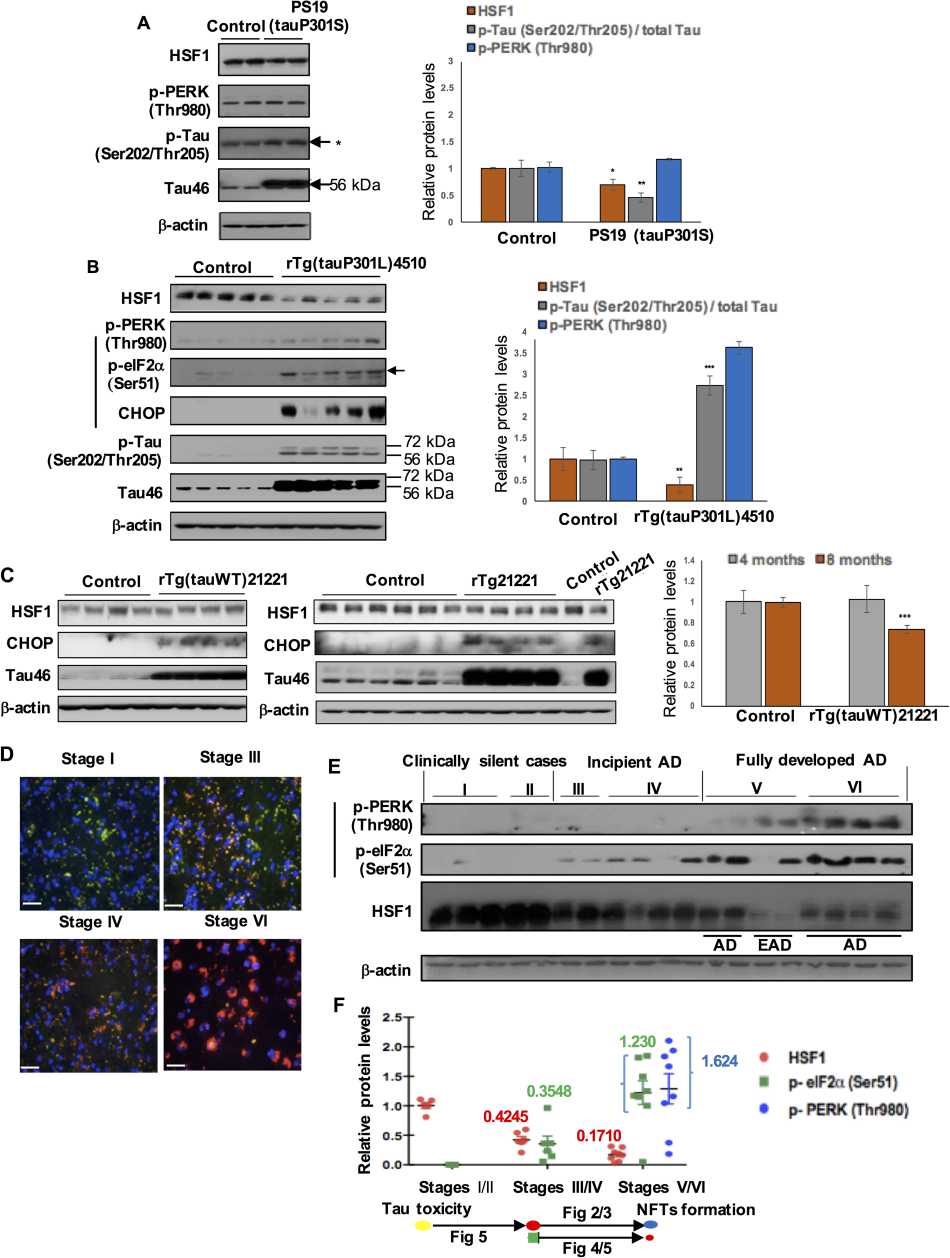
HSF1 protein loss and chronic UPR activation in the brains of human, rTg(tauP301L)4510, and rTg(tauWT)21221, and minor loss of HSF1 protein in the brain of PS19 (tauP301S). (A) Expression levels of HSF1, p-PERK (Thr980), total Tau (Tau46), and p-Tau (Ser202/Thr205) in PS19 (tauP301S) and WT at 4 months of age were determined by western blot. *: ~ 68 kDa tau isoform. (B) HSF1 loss and UPR activation in the brains of rTg(tauP301L)4510 at 4 months of age. Expression levels of HSF1, p-PERK (Thr980), p-eIF2α (Ser51), total Tau (Tau46) and p-Tau (Ser202/Thr205) in rTg4510 brain were determined by western blot (A vertical line highlights UPR marker proteins, herein after). Arrow indicates the band of p-eIF2α at 38 kDa, its original size. Tau46 antibody is expected to detect total Tau at 50–80 kDa. Control mice used are (from left to right) single transgenic (Tg) CaMKII-tTA, single Tg TRE-tau, non-Tg, non-Tg, and single Tg TRE-tau. Quantification of p-PERK, HSF1 (normalized to β-actin) and p-Tau (Ser202/Thr205) protein (normalized to total Tau protein (Tau46)) in the whole brain samples of PS19 (A), rTg4510 (B) and their control (means ± SEM, *P <0.05, **P <0.01, ***P <0.001, comparing to control, n = 5 (A), n = 10 (B)). (C) Expression levels of HSF1, CHOP, and total Tau (Tau46) in rTg(tauWT)21221 and control mice at 4- and 8 months of age. Quantification of HSF1 protein levels (normalized to β-actin) in the whole brain samples of 4- and 8 months old rTg21221 mice and their control (means ± SEM, ***P <0.001, comparing to control, n = 6 for each group (4 months), n = 7 (control, 8 months), n = 5 (rTg21221, 8 months)). Four month-old control mice used are (from left to right) two single transgenic Tg CaMKII-tTA and two single Tg TRE-tau. Eight month-old control mice used are (from left to right) two non-Tg, two single Tg CaMKII-tTA, and two single Tg TRE-tau. (D-F) HSF1 loss and UPR activation in the frontal cortex of human brain at different stages of Alzheimer's disease progression (Braak staging). (D) Tau aggregation and a dramatic loss of HSF1 protein in human AD brain of Braak stage VI. Phosphorylated tau (p-Tau (Ser202/Thr205), detected by AT8 antibody), red) and HSF1 protein expression (green) in four different Braak stages (Braak stage I, III, IV, VI) were visualized by immunostaining. Scale bar: 1 mm. (E) Protein lysates from frontal cortex of human brain at various Braak I-VI stages were subjected to western blot. EAD: early-onset Alzheimer's disease (AD). (F) Quantitative measurement of protein levels of p-PERK (blue circle), p-eIF2α (green square), and HSF1 (red circle) normalized to β-actin in human brain samples of distinct Braak stages (Braak stages I/II (Clinically silent cases); III/IV (Incipient AD); V/VI (Fully developed AD)). Error bars represent standard error of mean (SEM). Means indicated in the graph. The effect of left on right on each arrow is discussed in the following figures indicated.

To determine whether expression of HSF1 protein and UPR marker proteins was altered in the mouse tauopathy models, we looked at the brains of PS19 (tau P301S) mouse and rTg(tauP301L)4510 mouse overexpressing P301S and P301L mutant tau, respectively. It was previously reported that synaptic function was impaired in 3 month-old PS19 mouse before NFTs that consist of insoluble hyperphosphorylated tau developed at 6 months of age [25]. In the brain of 4 month-old PS19 mouse, we found that overexpressed mutant tau was not hyperphosphorylated at Ser202/Thr205 (p-Tau, detected using the AT8 antibody) (Fig 1A *P* < 0.01, n = 5). Upon activation of the UPR signal, PERK is phosphorylated at Thr980 (p-PERK). While PERK was not activated, about 30% of HSF1 protein was lost in the brain of PS19 mouse before tau hyperphosphorylation at a presumably later stage (Fig 1A, *P* < 0.05, n = 5). Insoluble tau filaments were previously detected in 4 month-old rTg4510 mouse [24]. We observed that overexpressed mutant tau was highly phosphorylated in the brain of rTg4510 mouse, about 2.7-fold increase in the levels of p-Tau normalized to total tau protein (Tau46), at 4 months of the same age with PS19 mouse above (Fig 1B, P < 0.001, n = 5). Activated PERK causes phosphorylation on Ser51 of the α subunit of eukaryotic translation initiation factor 2 (p-eIF2α). Together with p-PERK and p-eIF2α elevation, increased expression of pro-apoptotic protein CHOP suggested later apoptotic stage of UPR chronically activated in the brain of rTg4510 mouse (Fig 1B, p-PERK, P < 0.001, n = 5) [11, 12]. We detected a dramatic loss of HSF1 expression levels, about 60% reduction in rTg4510 mouse brain in which PERK (or UPR) was activated (Fig 1B, P < 0.01, n = 5). Furthermore, we looked at the brain of rTg21221 transgenic mouse that overexpresses non-aggregating wild-type human tau since no tau mutations have been identified in AD [28]. In the brain of rTg21221, while CHOP activation was induced at 4- and 8 months of age, about 30% of HSF1 protein was significantly lost at 8 months of age when compared to control group of mice (Fig 1C, P < 0.001, n = 7 (control), n = 5 (rTg21221)).

The Braak NFT staging system is used to classify the anatomical distribution of AD-type NFTs according to stages (I/II, entorhinal-; III/IV, limbic-; and V/VI, neocortical-stage) [29]. To identify the sequential relationship of molecular events in AD brain, we determined the expression levels of HSF1 protein and UPR marker proteins in the frontal lobes of 19 human postmortem brain specimen, spanning different Braak NFT stages. Distribution of HSF1 protein mostly in the cytoplasm suggested its inactivation in aged human brain since HSF1 is a nuclear transcriptional factor likely to be located in the nucleus when it is activated (Fig 1D). More importantly, we did observe gradually decreased expression of HSF1 protein that occurred even before formation of NFTs composed of hyperphosphorylated tau (Fig 1D–1F). While PERK activates eIF2α during UPR activation upon ER stress, other serine kinases such as general control nonderepressible 2 (GCN2) can also phosphorylate eIF2α in response to other stresses including amino acid deprivation. In incipient AD stages III/IV (before the frontal cortex starts to form NFTs), we observed about 58% reduction in HSF1 protein and slightly increased p-eIF2α without PERK activation (Fig 1E and 1F). UPR was activated in later stage V patients diagnosed with early onset AD (EAD) and AD patients in stage VI, as reflected by marked hyperactivation of PERK and eIF2α (Fig 1E and 1F). About 59% of total HSF1 protein in Stages III/IV was further lost in stages V/VI when UPR was activated. It should be noted that stage V patients diagnosed with EAD demonstrated significantly greater p-PERK and less HSF1 protein expression when compared to AD patients in stage V (Fig 1E). Taken together, these results identify that a dramatic loss of HSF1 protein is an early and progressive event that may precede PERK activation and NFTs formation in murine tauopathy and human AD.

### Haploinsufficiency of HSF1 leads to chronic PERK activation and causes aberrantly phosphorylated tau aggregation and its mislocalization in the aged mouse hippocampus

**Fig 2:**
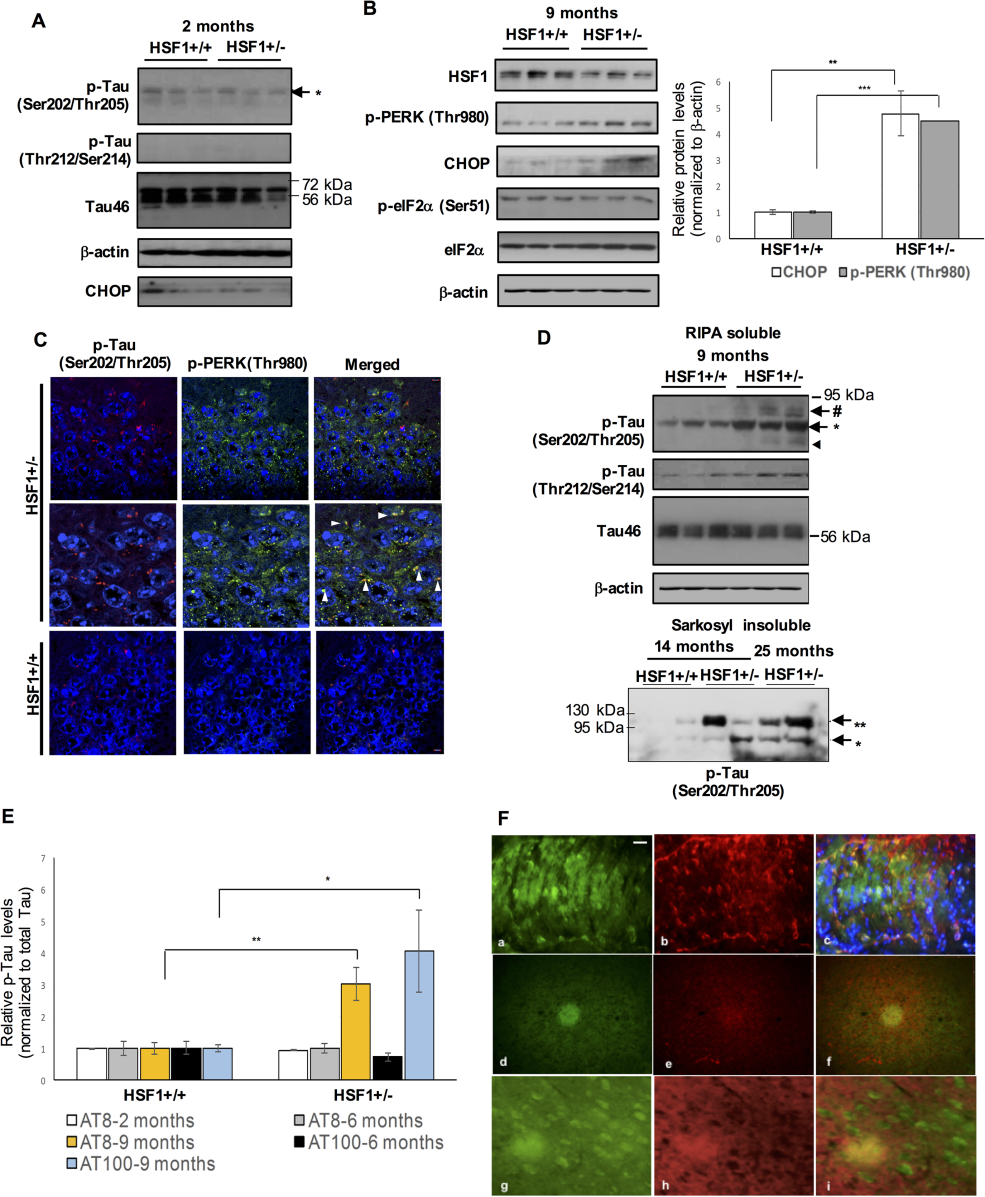
Chronic UPR activation and aberrantly hyperphosphorylated tau aggregation in the hippocampus of aged HSF1 haploinsufficient mouse. (A, B, and D) Western blot assays to examine protein expression levels of total tau (Tau46), p-Tau (at Ser202/Thr205 and Thr212/Ser214) and UPR marker proteins (p-PERK, p-eIF2α, eIF2α, and CHOP) in the hippocampus of WT (HSF1+/+) and HSF1+/- at 2 months of age (A) and hippocampus of WT and HSF1+/- at 9 months of age (B, D). (B) Increased p-PERK (Thr980) and CHOP protein expression in the hippocampus of 9 month-old HSF1 heterozygous knock-out mice (HSF1+/-). Relative protein levels of CHOP and p-PERK (Thr980) normalized to β-actin in WT and HSF1 +/- at ~9 months of age are presented in the graph (right, means ± SEM, **P < 0.01, ***P < 0.001, n = 6). Western blots of RIPA-soluble tau (A and D) and sarkosyl-insoluble tau aggregates (D) in the mouse hippocampus. #: 72~80 kDa tau isoform, *: ~ 68 kDa tau isoform, arrow head indicates ~64 kDa tau isoform, **: ~110 kDa high molecular weight tau isoform. (C) Representative confocal images of immunohistochemistry on the hippocampus of HSF1 +/- and WT at 13 months of age. co-immunostaining of p-Tau (Ser202/Thr205, red), p-PERK (Thr980) (green), and DAPI (blue) in HSF1+/-. Arrow heads indicate co-localization of p-PERK and p-Tau (Ser202/Thr205) in the middle panels, enlarged images of top panels. Five repeated experiments performed. Scale bars: 5 μM. (E) A graph shows quantitative measurement of the relative tau phosphorylation normalized to total tau (Tau46) in the hippocampus of WT and HSF1+/- at 2, 6, and 9 months of age. Two distinct p-Tau antibodies (AT8 (Ser202/Thr205) and AT100 (Thr212/Ser214)) used. (means ± SEM, *P < 0.05, **P < 0.01, n = 3 (2 months), n = 4 (6 months) n = 6 (9 months)). (F) Double staining with thioflavin S (green, a) and Tau46 antibody (red, b) in the hippocampus of 13 month-old HSF1 +/-. Merged images of the hippocampus of HSF1+/- (c). Globose-type NFTs and diffuse plaques containing tau aggregates in the cortical areas adjacent to the hippocampus of HSF1+/- (d-i). Scale bar: 1 mm.

Next, we investigated if HSF1 protein loss altered the expression levels of UPR marker proteins and tau phosphorylation in the brain of HSF1 haploinsufficient mouse (HSF1+/-). Phosphorylation at Thr212/Ser214 (p-Tau, detected using the AT100 antibody) was not detected in the hippocampus of 2 month-old HSF1+/- and WT (Fig 2A). We did not observe any significant change in the expression levels of p-Tau and CHOP in the hippocampus of HSF1+/- at 2- and 6-months of age (Fig 2A and 2E and S1A Fig). However, PERK and CHOP protein were highly activated in the hippocampus of 9 month-old HSF1+/-, although p-eIF2α was not elevated (Fig 2B, p-PERK (Thr980), *P* < 0.001; CHOP, *P* < 0.01, comparing to 9 month-old WT hippocampus, n = 6). By immunohistochemistry, we found that activated PERK (p-PERK, green) largely co-localized with of increased p-Tau (Ser202/Thr205, red) in the hippocampus of HSF1+/- at 13 months of age (Fig 2C). Of note, a dramatic upregulation of p-Tau (detected by both AT8 and AT100 antibodies) was strongly found in the hippocampus of 9 month-old HSF1+/- by western blot (Fig 2D and 2E, p-Tau (Ser202/Thr205), *P* < 0.01; p-Tau (Thr212/Ser214), *P* < 0.05, n = 6). These changes were not detectable in the whole brain lysates of 9 month-old HSF1 +/- (S1B Fig). Tau isolated from PHFs in human AD brain has been reported to contain ~60-, ~64-, and ~68- kDa tau isoforms [30]. In our study, the strongest immunoreactive band for phosphorylated tau protein in HSF1+/- was detected at ~68 kDa (Fig 2D). In the aged HSF1+/- hippocampus, there were two major p-Tau bands at ~64- and ~68- kDa with a minor band at ~72 kDa, similar to the pattern observed in human tauopathy brain [31] (Fig 2D). These are considered to be low molecular weight tau, ranging in 50–68 kDa on SDS-PAGE [32, 33]. Sarkosyl insolubility assay is used to isolate tau paired helical filaments found in NFTs from AD brain [34]. In the sarkosyl-insoluble fraction from the hippocampus of aged HSF1+/-, we found high molecular weight tau isoform, approximately 110 kDa tau, in addition to a major band of ~68 kDa [35] (Fig 2D and S1C Fig). Paired helical filaments from AD brain bind to Thioflavin S [36]. By double staining using thioflavin S and Tau antibody (Tau46), aggregates of hyperphosphorylated tau were found in the hippocampus of 13 month-old HSF1+/-, not in WT control (Fig 2F and S2 Fig). Diffuse plaques that showed strong positive signals for thioflavin S consisted of tau proteins (Fig 2F). Thioflavin S staining also revealed globose-type NFTs and diffuse plaques in the cortical areas adjacent to the hippocampus (Fig 2F).

**Fig 3:**
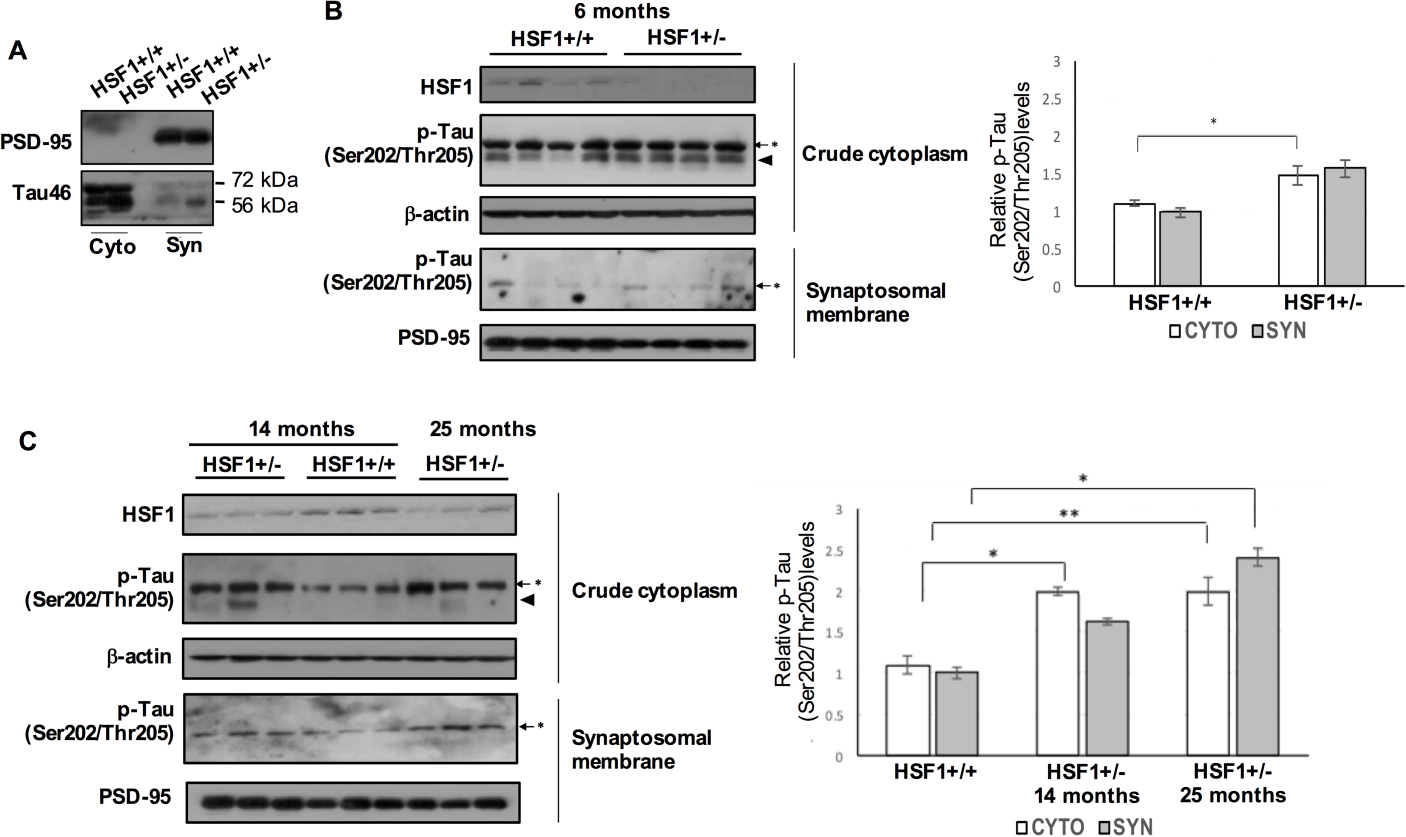
Abnormal mislocalization of hyperphosphorylated tau in the hippocampus of HSF1 haploinsufficient mouse. (A) PSD-95 highly enriched in the synaptosomal membrane fraction of 13 month-old mouse hippocampus. Tau46 antibody was used to detect total tau protein in the crude cytoplasm and synaptosomal membrane fraction. (B and C) Increased expression of p-Tau (Ser202/Thr205) in the cytoplasm and synaptosomal membrane fraction from the hippocampus of HSF1+/-. Graphs indicate quantification of p-Tau (Ser202/Thr205) protein levels normalized to β-actin (cytoplasm) and PSD-95 (synaptosomal membrane) in the hippocampus of WT and HSF1+/- at different ages as indicated (means ± SEM, *P < 0.05, **P < 0.01, n = 4 (B), n = 3 (C)).

Although tau protein is most abundant in axons, abnormal modifications of tau such as hyperphosphorylation can lead to redistribution of tau from neuronal processes to the soma where it likely forms toxic oligomers or aggregates [37]. Hyperphosphorylation of tau has been identified to mislocalize tau to dendritic spines in neurons in AD models [28]. Mouse hippocampal tissues were processed by crude cytoplasmic extraction and synaptosomal fractionation that enriches both presynaptic and postsynaptic compartments. Both cytoplasmic- and synaptic- tau were found to increase in the hippocampus of 13 month-old HSF1+/- compared to WT control hippocampus (Fig 3A). We found increase in the average of the relative expression of p-Tau (Ser202/Thr205) in the cytoplasm and synaptosomal membrane of the hippocampus of 6 month-old HSF1+/- compared to that of 6 month-old WT (Fig 3A, cytoplasmic p-Tau (Ser202/Thr205), *P* < 0.05). Clearly, expression levels of p-Tau (Ser202/Thr205) were significantly upregulated in the both cytoplasm and synaptosomal membrane of the hippocampus of 14- and 25- month-old HSF1+/- compared to that of 14 month-old WT (Fig 3B).

### eIF2α-CHOP activation is involved in autophagy-lysosomal HSF1 protein degradation during chemically induced ER stress in neurons.

**Fig 4:**
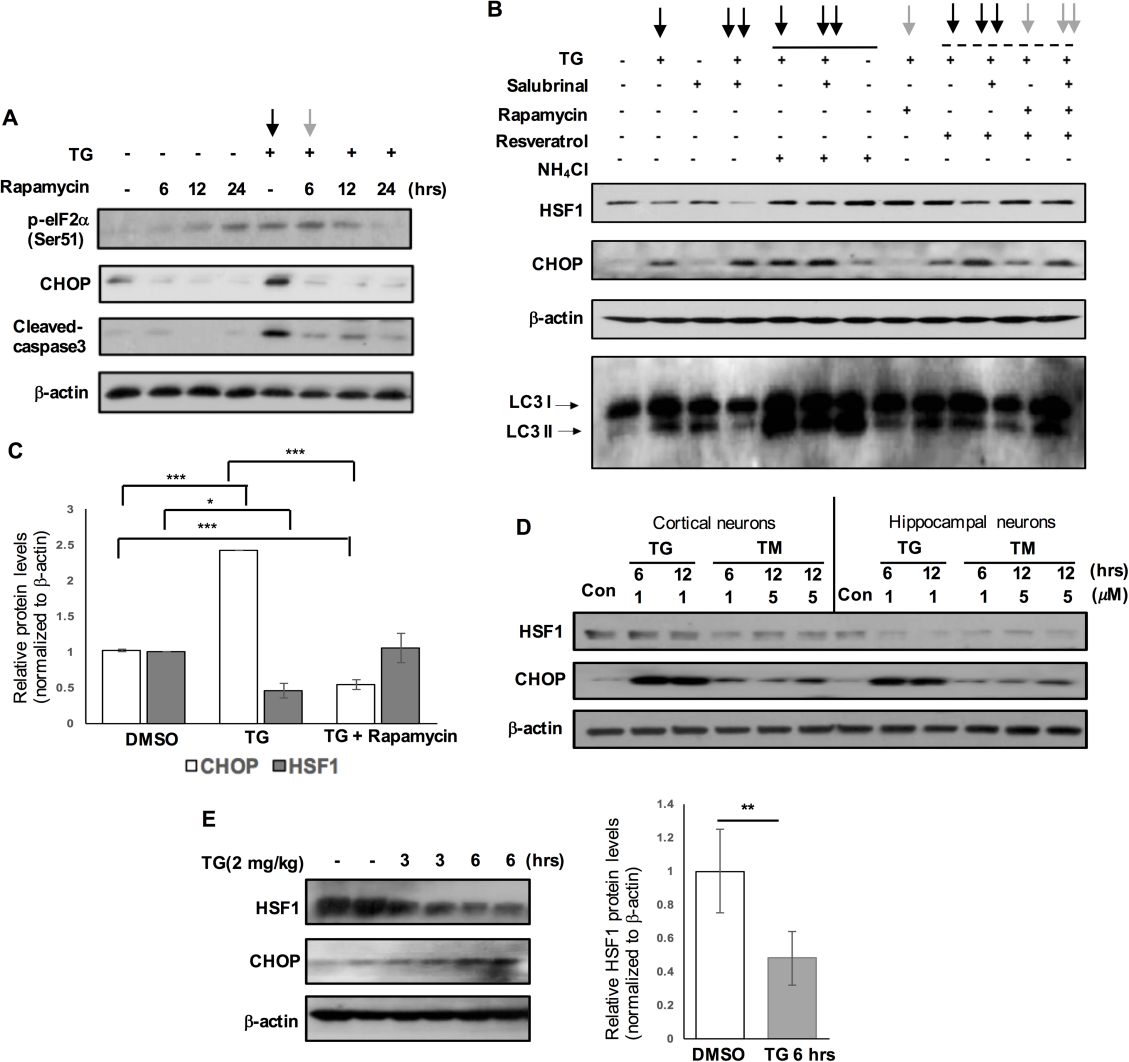
Autophagy-lysosomal HSF1 protein degradation mediated by eIF2α-CHOP activation during chemically induced acute ER stress in neurons. (A) Reduced pro-apoptotic CHOP activation by rapamycin treatment during thapsigargin (TG)-induced ER stress. Primary cortical neurons were pre-treated with rapamycin (0.4 μM, for indicated duration time) followed by TG incubation (1 μM, the last 6 hr). Cleaved-caspase 3 as apoptotic marker. (B) The combination treatments of ER stress-inducer (TG, 1 μM), ER stress-inhibitor (rapamycin, 0.5 μM), p-eIF2α activator (salubrinal, 20 μM), lysosomal blocker (NH_4_Cl, 5 mM) and resveratrol (5 μM) for 6 hr in primary cortical neurons to study the relationship between HSF1 protein and eIF2α-CHOP activation. LC3 II increase by TG and NH_4_Cl (LC3 I and II (autophagy markers)). ↓: thapsigargin, ↓: co-treatment of thapsigargin and rapamycin, ↓↓: co-treatment of thapsigargin and salubrinal, A solid and a dashed line indicate the treatments of NH_4_Cl and resveratrol, respectively. (C) A graph represents quantification of relative protein levels of HSF1 and CHOP in the three distinct groups of primary cortical neurons (means ± SEM, *P <0.05, ***P <0.001, n = 3–4). TG (1 μM, 6 hr) and rapamycin (0.5 μM, 6 hr), (D) Rat primary cultured cortical and hippocampal neurons were treated with tunicamycin (TM) and TG at different doses (1 μM and 5 μM) and duration times (6 hr and 12 hr). (E) Cerebral cortex of 5 month-old B6 mice subjected to the intraperitoneal injection of thapsigargin (TG, 2 mg/kg) were harvested at different time points indicated and subjected to western blot. A graph showing the change on the relative protein levels of HSF1 in the mouse cortex after TG treatment (**P < 0.01, comparing to control injected with PBS containing 10% DMSO, means ± SEM, n = 5).

We observed that loss of HSF1 protein was more severe in the brains of rTg4510 and Braak stages V/VI subjects where PERK was activated when compared to that of age-matched PS19 and stages III/IV subjects that did not show PERK activation, respectively (Fig 1). Thus, we asked if UPR activation was involved in HSF1 protein loss in rat primary cultured neurons. Thapsigargin triggers ER stress by inhibiting Ca2+-transporting, ATPase mediated uptake of calcium ions into the sarcoplasmic reticulum. It is well documented that rapamycin, an inhibitor of mTOR (mammalian target of rapamycin), represses ER stress in various cell types [38]. Rapamycin seemed to act as a reliable inhibitor of CHOP expression both in the presence and absence of thapsigargin in primary cortical neurons (Fig 4A–4C, ↓, P < 0.001, n = 3–4). We demonstrated that thapsigargin triggered pro-apoptotic UPR activation that was attenuated by co-treatment of rapamycin (Fig 4A–4C). Of note, thapsigargin treatment led to ~54% reduction in the HSF1 protein levels in primary neurons (P < 0.05, n = 4), which was nearly blocked by rapamycin (Fig 4B and 4C). CHOP activation could be further enhanced by salubrinal treatment which inhibits activity of protein phosphatase 1 and prolongs eIF2α phosphorylation. In contrast to rapamycin, salubrinal treatment further aggravated HSF1 loss from thapsigargin-induced ER stress in primary neurons (Fig 4B, ↓↓). Autophagy activity, as determined by LC3 II accumulation, was increased in thapsigargin-treated neurons (Fig 4B, ↓). Treatment of autophagy-lysosomal blocker, NH_4_Cl, inhibited thapsigargin-induced HSF1 loss, revealing HSF1 protein degradation through autophagy-lysosomal system (Fig 4B, a solid line indicated). Although direct HSF1 activator has not been identified, we previously reported that resveratrol could prevent HSF1 degradation from proteotoxic stress [19]. In the current study, resveratrol attenuated thapsigargin-induced HSF1 protein degradation in primary neurons (Fig 4B, a dashed line indicated). Tunicamycin is another chemical UPR inducer that causes protein unfolding by blocking the glycoprotein synthesis pathway. Tunicamycin also reduced HSF1 protein expression in primary cultured cortical and hippocampal neurons (Fig 4D). We then sought *in vivo* evidence of thapsigargin-induced HSF1 degradation. Five month-old C57BL/6 mice were injected with thapsigargin (i.p. 2 mg/kg) or PBS containing 10% DMSO. Remarkably, 6 hrs later, drastic HSF1 degradation, about 50% reduction in HSF1 protein expression, was detected in the mouse brain injected with thapsigargin (Fig 4E, P < 0.01, n = 5).

### Overexpressed pro-aggregation mutant Tau_RD_ ΔK280 leads to HSF1 degradation which is further exacerbated by eIF2α-CHOP activation

**Fig 5:**
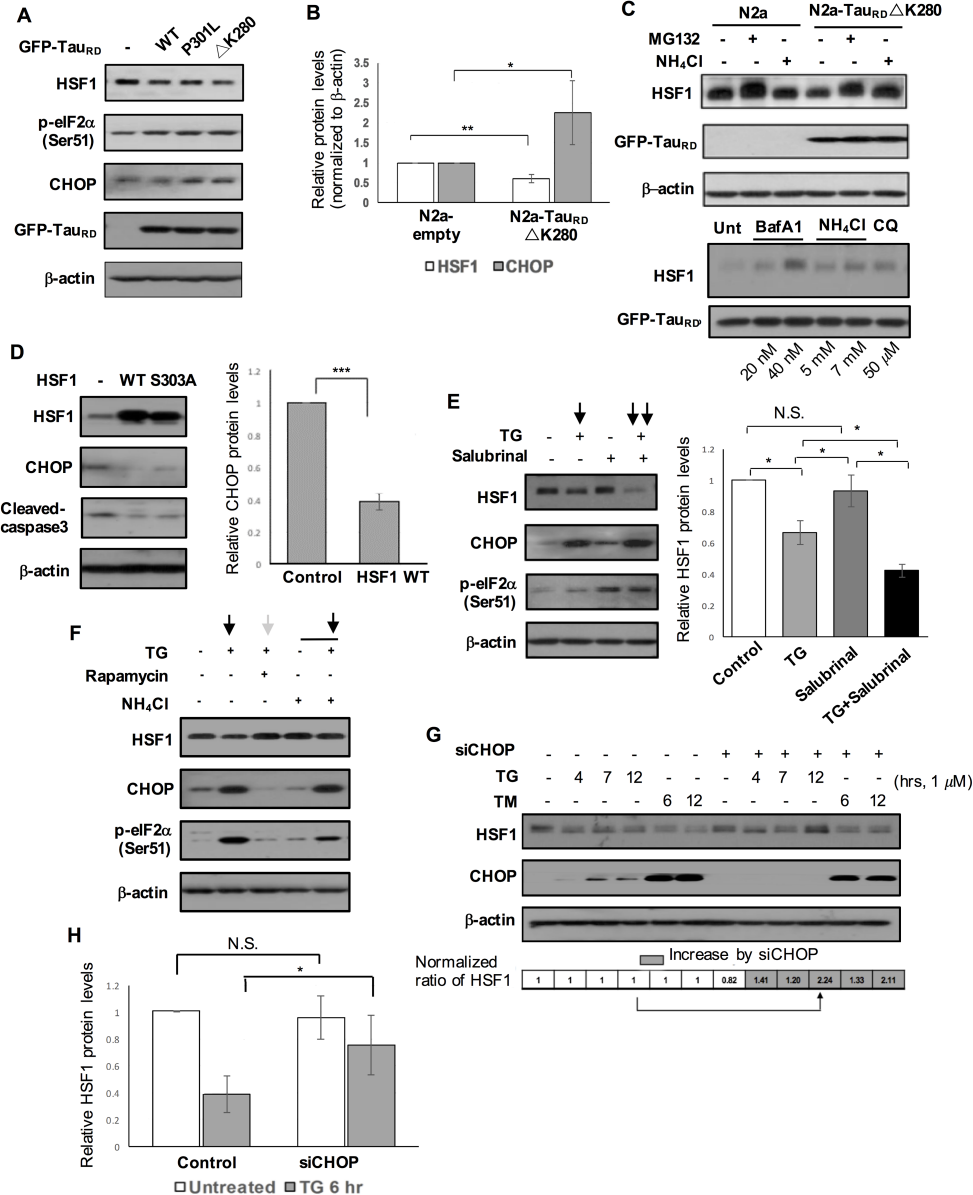
HSF1 degradation caused by overexpressed Tau_RD_ ΔK280 and further facilitated by eIF2α-CHOP activation. (A-C) HSF1 degradation and CHOP activation in N2a-Tau_RD_ ΔK280 stable cell line. (A) Western blot analysis on N2a neuroblastoma cells transiently overexpressing GFP-tagged repeated domain (RD) tau constructs (Tau_RD_ WT, Tau_RD_ P301L, Tau_RD_ ΔK280) and empty vector (control). (B) A graph indicates relative protein levels of HSF1 and CHOP normalized to β-actin in N2a and N2a-Tau_RD_ ΔK280 stable cell line (means ± SEM, *P <0.05, **P <0.01, comparing to control, n = 4). (C) HSF1 degradation through both autophagy-lysosome and proteasome in N2a-Tau_RD_ ΔK280. N2a-Tau_RD_ ΔK280 were treated with either autophagy-lysosomal (Baf A1, NH_4_Cl, CQ) or proteasomal blocker (MG 132, 20 μM) for 6 hr at different concentrations indicated. Left: Control N2a treated with either MG132 or NH_4_Cl. Right (short exposure of film): Baf A1: baflomycin A1, CQ: chloroquine. (D) N2a-Tau_RD_ ΔK280 were transfected with either HSF1 WT or HSF1 S303A (constitutively active form) or empty vector as control. Cleaved-caspase 3 as apoptotic marker. A right graph represents reduced relative CHOP protein expression levels normalized to β-actin in N2a-Tau_RD_ ΔK280 overexpressing HSF1 WT (means ± SEM, ***P < 0.001, comparing to N2a-Tau_RD_ ΔK280 transfected with empty vector, n = 4). (E) N2a-Tau_RD_ ΔK280 were treated with either thapsigargin (TG, 1μM) for 6 hr or salubrinal (20 μM) for 12 hr or both of them. Their protein lysates were subjected to western blot (left). A graph on quantitative measurement of the relative HSF1 protein expression (right, means ± SEM, *P < 0.05, n = 4, N.S., non-significant). (F) Treatment of thapsigargin (1 μM), rapamycin (0.5 μM) and NH_4_Cl (5 mM) in N2a-Tau_RD_ ΔK280. (G) The change on expression levels of HSF1 was assessed in N2a-Tau_RD_ ΔK280 transfected with CHOP siRNA and later subjected to either thapsigargin or tunicamycin (1 μM) treatment for different duration time as indicated. Quantitative measurement of relative HSF1 protein levels normalized to β-actin (bottom, arrow indicates dramatica increase in HSF1 protein by siCHOP expression.) (H) A graph indicates relative HSF1 protein levels in N2a-Tau_RD_ ΔK280 transfected with CHOP siRNA in the presence or absence of thapsigargin (means ± SEM, *P < 0.05, N.S., non-significant, n = 3).

Loss of HSF1 protein began to occur even in the absence of PERK activation in Braak stage III/IV and PS19 mouse (Fig 1). There is a possibility that proteotoxic stress from pathogenic tau affected HSF1 expression [19]. To determine if overexpressed mutant tau caused HSF1 protein loss and eIF2α-CHOP activation *in vitro*, we transiently transfected N2a neuroblastoma cells with wild-type- or P301L- or ΔK280-Tau_RD._ Tau protein typically does not form amyloid fibrils *in vitro* because of its intrinsic hydrophilic feature. The lack of amyloidogenic propensity can be overcome by using the tau repeated domain (Tau_RD_), the most commonly used form of truncated tau. The four conserved sequence motifs in this domain are essential for tau aggregation. Both eIF2α-CHOP activation and decreased HSF1 protein expression were manifested in ΔK280 Tau_RD_ transfected N2a cells (Fig 5A). Thus, we generated N2a cells stably overexpressing Tau_RD_ ΔK280 (N2a-Tau_RD_ ΔK280) as a cellular model to study the relationship of CHOP activation and HSF1 loss in the following studies. In N2a-Tau_RD_ ΔK280, about 40% of HSF1 protein was lost whereas 2.3-fold increase in CHOP protein was found in N2a-Tau_RD_ ΔK280, which was statistically significant (Fig 5B, HSF1, P < 0.01; CHOP, P < 0.05, n = 4). We observed a remarkable HSF1 increase after treatment with each of three agents to inhibit autophagy-lysosome pathway and MG132, a proteasomal blocker, suggesting that HSF1 protein was degraded by both UPS and autophagy-lysosome in N2a-Tau_RD_ ΔK280 (Fig 5C).

Since reduced HSF1 expression caused CHOP activation in the mouse brain (Fig 2), we asked if HSF1 degradation was related with CHOP activation in N2a-Tau_RD_ ΔK280. Overexpressed HSF1 WT reduced about 60% of CHOP protein expression in N2a-Tau_RD_ ΔK280 (Fig 5D, *P* < 0.001, n = 4). Conversely, eIF2α-CHOP activation seemed to further promote HSF1 degradation in N2a-Tau_RD_ ΔK280 (Fig 5E, P < 0.01, n = 4), as seen in primary neurons in Fig 4. Suppression of CHOP activation by rapamycin attenuated thapsigargin-induced autophagy-lysosomal HSF1 degradation in N2a-Tau_RD_ ΔK280 (Fig 5F). During thapsigargin and tunicamycin treatments for various time periods, reduction of CHOP expression via siRNA upregulated HSF protein expression in N2a-Tau_RD_ ΔK280 (Fig 5G), which was statistically significant (Fig 5H, P < 0.05, n = 4). However, in the absence of thapsigargin, CHOP was revealed not to be a primary component in HSF1 degradation present in N2a-Tau_RD_ ΔK280, as confirmed by the lack of statistically significant HSF1 change following CHOP silencing (Fig 5H).

### Aberrant HSF1 degradation and HSP70 a5 (BiP/GRP78) attenuation are associated with tau accumulation and toxicity in tauopathy

**Fig 6:**
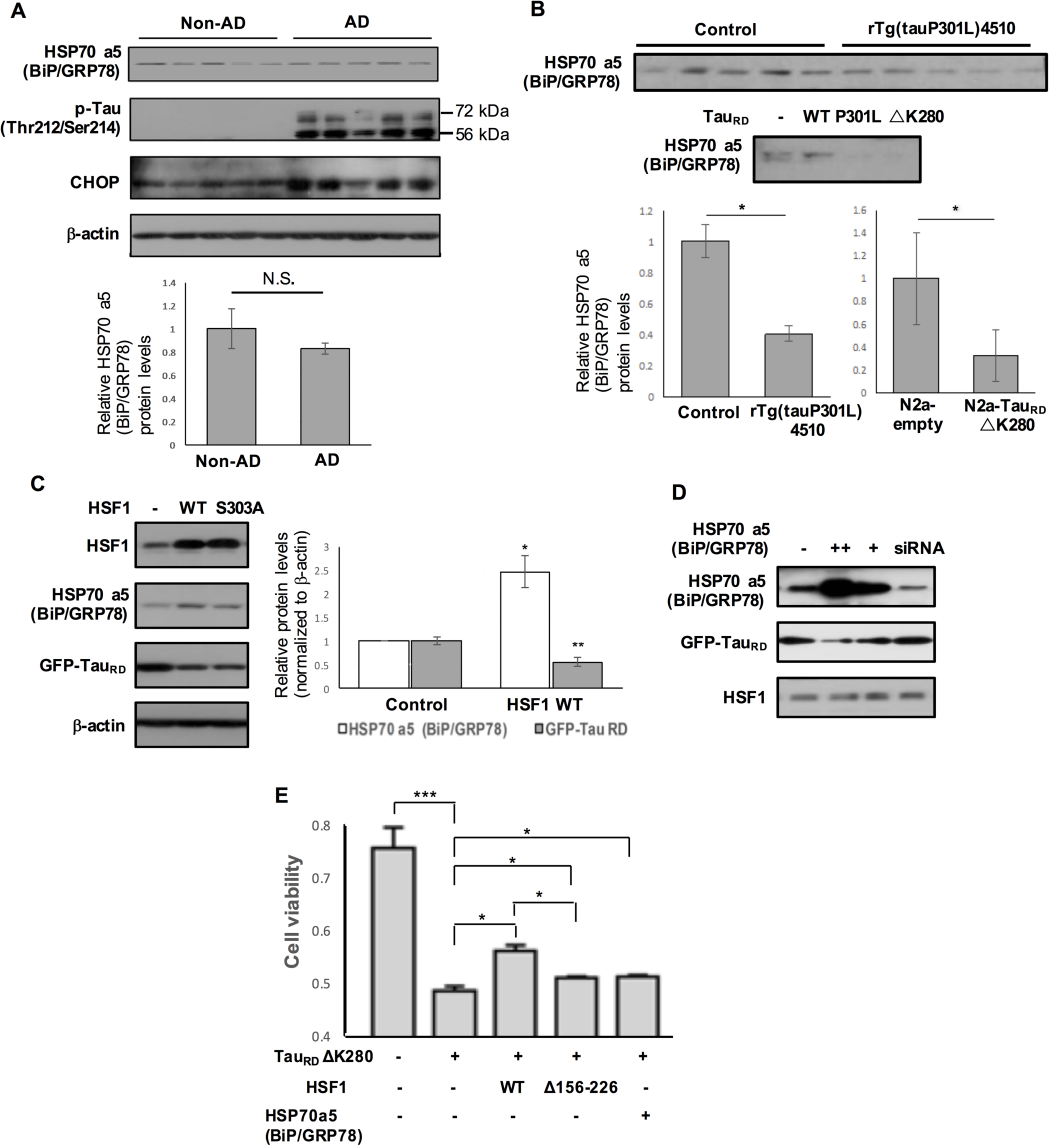
The involvement of aberrant HSF1 degradation and HSP70 a5 (BiP/GRP78) attenuation in tau accumulation and toxicity in tauopathy. (A and B) Reduced HSP70 a5 (BiP/GRP78) expression common in human AD brains (A) and cellular and mouse tauopathy models (B). Graphs show reduction in relative HSP70 a5 (BiP/GRP78) protein expression normalized to β-actin in N2a-Tau_RD_ ΔK280 (n = 4, * *P* < 0.05), human AD brains (n = 5, non-significant) and rTg(tauP301L)4510 (n = 5, * *P* < 0.05). Data represents means ± SEM. Β–actin for Fig 6B was shown in Figs 1B and 5A. (C and D) N2a-Tau_RD_ ΔK280 were transfected with either HSF1 WT or HSF1 S303A (constitutively active form) (C) or HSP70 a5 (BiP/GRP78) or BiP siRNA (D). Control N2a-Tau_RD_ ΔK280 transfected with empty vector. (C) A right graph on the relative HSP70 a5 (BiP/GRP78) and GFP-Tau_RD_ protein expression levels normalized to β-actin in N2a-Tau_RD_ overexpressing HSF1 WT (means ± SEM, *P < 0.05, HSP70 a5 (Bip/GRP78); **P < 0.01, GFP- Tau_RD_, comparing to control, n = 4). (D) Reduced GFP-Tau_RD_ accumulation by HSP70 a5 (BiP/GRP78) overexpression in N2a-Tau_RD_ ΔK280. (E) Cell viability in N2a-Tau_RD_ ΔK280 overexpressing either HSF1 WT or HSF1 Δ156–226, or HSP70 a5 (BiP/GRP78) and control was measured by MTT assay (means ± SEM, *P < 0.05, n = 5).

**Fig 7:**
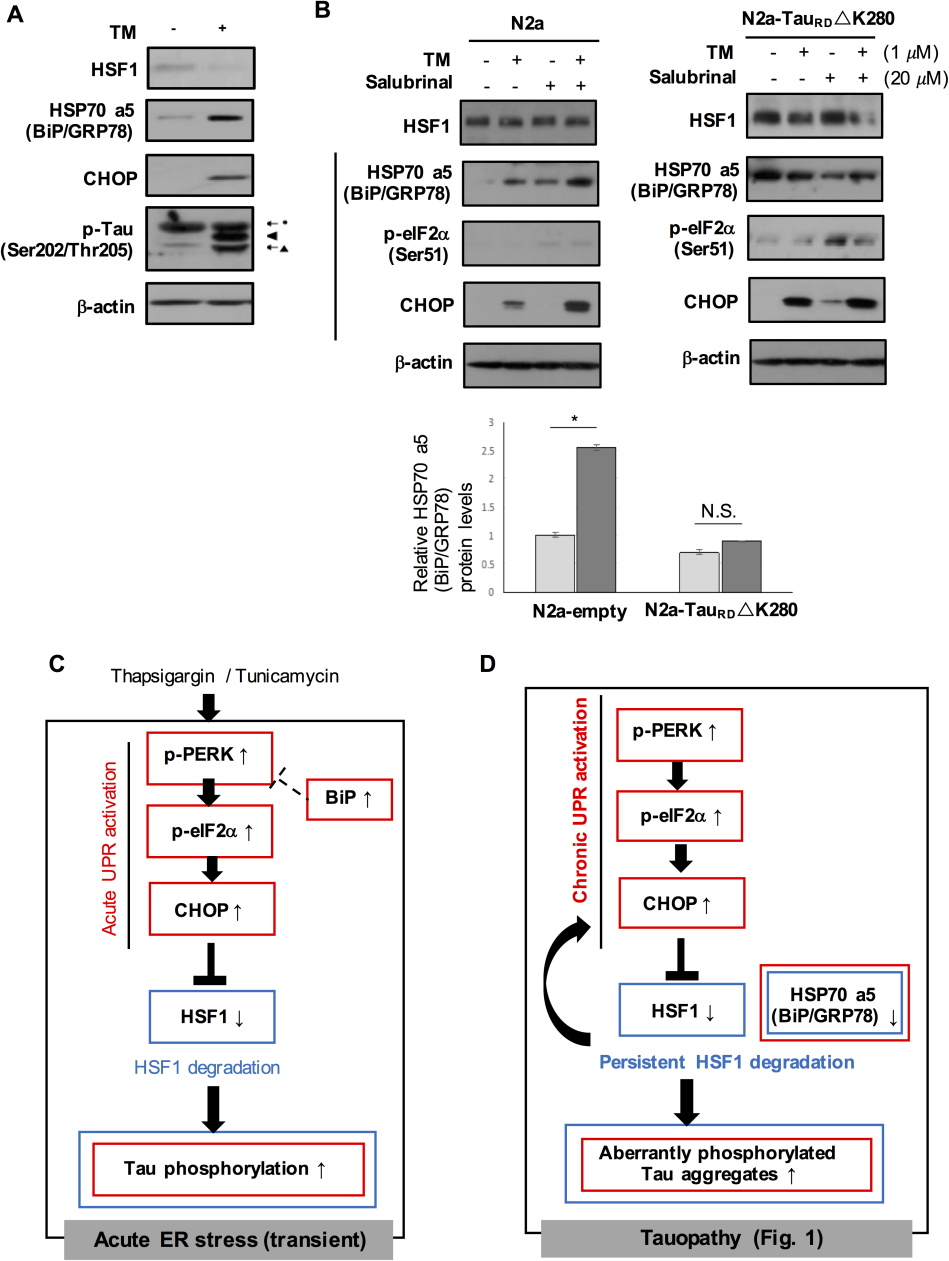
Suppressed induction of HSP70 a5 (BiP/GRP78) upon chemical ER stress in N2a-Tau_RD_ ΔK280 and schematic representation of the hypothetic model on a vicious cycle of UPR activation and HSF1 degradation in tauopathy. (A) Primary cortical neurons treated with tunicamycin (TM, 1 μM). (B) N2a-Tau_RD_ ΔK280 and control N2a cells were treated with either tunicamycin (TM, 1 μM) for 6 hrs or salubrinal (20 μM) for 12 hrs or both of them. Their protein lysates were subjected to western blot. A right graph on quantitative measurement of the relative HSP70 a5 (BiP/GRP78) protein expression in response to TM treatment in N2a-Tau_RD_ ΔK280 and control N2a (means ± SEM, *P < 0.05, N.S., non-significant, n = 4). (C) Acute ER stress activates both CHOP and BiP proteins, which may ‘transiently’ lead to HSF1 degradation (Fig 4) and tau phosphorylation. Activated BiP may contribute to cease UPR activation [9]. (D) Chronic UPR activation (CHOP activation and BiP attenuation) in tauopathy. Persistent HSF1 loss seemed to cause chronic CHOP activation (Figs 2 and 3), starting to form a vicious cycle of chronic CHOP activation and persistent HSF1 degradation. This eventually may lead to the formation of aberrantly phosphorylated tau aggregates in the aged hippocampus (Figs 2 and 3). HSP70 a5 (BiP/GRP78) attenuation may contribute to tau accumulation and its toxicity in tauopathy (Fig 6). (Red, UPR-related proteins; Blue, HSF1-regulated proteins).

HSP70 a5 (BiP/GRP78), a major ER chaperone protein, acts as a negative regulator of UPR signaling [9, 39]. The promoter of HSP70a5 possesses DNA sequences called heat shock elements where HSF1 can bind for transcriptional activation [40]. In contrast to CHOP elevation, HSP70 a5 (BiP/GRP78) expression normalized to β-actin was slightly reduced in the human AD brains when compared to non-AD brains (Fig 6A, non-significant). In addition, the steady state level of HSP70 a5 (BiP/GRP78) was 70% less and 60% less in N2a-Tau_RD_ ΔK280 and rTg(tauP301L)4510 than their control, respectively (Fig 6B). Overexpressed HSF1 protein in N2a-Tau_RD_ ΔK280 not only significantly enhanced HSP70 a5 (BiP/GRP78) expression but also remarkably reduced Tau_RD_ΔK280 (Fig 6C, HSP70 a5 (BiP/GRP78), P < 0.05; GFP-Tau_RD_, P < 0.01, n = 4). Overexpressed HSP70 a5 (BiP/GRP78) reduced Tau_RD_ ΔK280 accumulation without affecting HSF1 protein expression in N2a-Tau_RD_ ΔK280 (Fig 6D). These enhanced expression of HSF1 and HSP70 a5 (BiP/GRP78) led to increased cell survival in N2a-Tau_RD_ ΔK280 (Fig 6E). Overexpressed HSF1 mutant (i.e. HSF1Δ156–226) deficient in trimerization of a prerequisite step for transcriptional activation still demonstrated increased cell viability, however to a lesser degree than observed in HSF1 WT (Fig 6E and S4 Fig). In primary neurons and N2a cells (Fig 7A and 7B), in response to tunicamycin treatment, protective UPR was activated that increased expression of HSP70 a5 (BiP/GRP78) (Fig 7A and 7B, P < 0.05, n = 4). In contrast, this protective stress response was not elicited in our cellular tauopathy model of N2a-Tau_RD_ ΔK280, supporting attenuated HSP70 a5 (BiP/GRP78) expression in tauopathy (Fig 7A and 7B, n = 4).

## Discussion

Here we provide both *in vitro* and *in vivo* evidence that strongly suggests an auto-propagating interplay of UPR activation and HSF1 degradation being a common pathogenic feature in both human AD and tau transgenic mouse AD models (Fig 7). Characterizations of ER stress on early-stage AD / MCI patients have been neglected in many studies. The underlying mechanisms leading to chronically sustained ER stress in human AD brain are not entirely understood. This experiment looks to identify persistent and striking HSF1 degradation as an integral component in the chronic UPR activation pathway that ultimately causes tau hyperphosphorylation in early AD pathogenesis.

It should be noted that proteotoxic stress from tau aggregation could promote de-stabilization of HSF1 protein in several ways that are not clearly understood (Figs 1 and 5). We implicate CHOP activation in the UPR pathway as one of key players that partially contributes to HSF1 degradation in tauopathy (Figs 4 and 5). CHOP activation can be regulated in the ER not only by PERK-eIF2α but also by IRE1 or other unknown ER-independent mechanism [38]. Consequently, reduced steady state level of HSF1 is likely to trigger permanent PERK-CHOP activation (Fig 2) that reversely facilitates HSF1 loss, leading to tau hyperphosphorylation in tauopathy (Figs 2 and 7D). It can therefore be reasonably concluded that suppression of pro-apoptotic protein CHOP could be an efficient means of protecting HSF1 from ER stress-related tauopathy. However, it was also revealed that in the young HSF1+/- mouse brain, HSF1 depletion alone was insufficient to cause either ER stress or tau phosphorylation (Fig 2), but another unidentified aging-related pathway with which HSF1 degradation associates with may actually cause disturbances in ER homeostasis. The relevant kinase(s) affecting tau phosphorylation in CHOP-HSF1 axis should also be determined in further studies. In addition to the role of autophagy-lysosomal activity on the HSF1 protein turnover, given highly poly-ubiquitinated HSF1 protein in N2a-Tau_RD_ ΔK280 (S5B Fig), it is necessary to investigate the involvement of ubiquitin-proteasome system in tauopathy as we did in synucleinopathy [19]. Tau toxicity can impair another essential response in ER called Endoplasmic-reticulum-associated protein degradation (ERAD) [26]. Hrd1 is an ERAD-associated E3 ubiquitin protein ligase previously found to interact with tau [26]. We observed a tremendous decrease in Hrd1 expression in rTg4510 (S6 Fig). The potential interactions between ERAD and HSF1 should be considered in future studies.

Considering HSF1 loss observed in Braak III/IV stages, it also leaves open the possibility of the involvement of amyloid pathology in HSF1 degradation. We found that the extent of HSF1 loss was more severe in tau transgenic mice (Tg4510) than in APP transgenic mice (Tg2576) that produced amyloid beta plaques [41] (Fig 1D). Tg2576 mice were reported not to show any signs of UPR activation by others [42]. However, the more recent *in vitro* data suggests possible involvement of UPR activation in Aβ toxicity [43]. It is thus necessary to identify whether Aβ can cause ER stress *in vivo*, and if so, whether or not this mechanism is tau-dependent.

Another corollary of HSF1 loss represented in the study is that it can be deterministic of the balance between pro-apoptotic (CHOP) and pro-survival responses (BiP) to ER stress. Inhibited HSP70 a5 (BiP/GRP78) protein expression is consistently observed in all our tauopathy models (N2a-Tau_RD_ΔK280, rTg(taupP301L)4510, and human AD, Fig 6). It may lead to an inability to attenuate UPR [9, 39], contributing to tau aggregation and its toxicity in tauopathy (Fig 6). *In vivo* studies on Tau ΔK280 transgenic mice indicated that tau toxicity was closely related to its ability to form aggregates [23]. We could garner some evidence to speculate that the highly aggregated fibrillary form of tau is the major causative species to induce HSF1 degradation. Overexpressed full-length Tau WT, Tau P301L, and Tau R406W in N2a cells did not significantly alter HSF1 protein levels (S5A Fig). Each tau repeated domain only (Tau_RD_) and the mutant tau transgene lacking N-terminal insert (expressed in rTg4510) are all considered to aggregate faster than full length tau and the mutant tau transgene including one N-terminal insert (expressed in PS19), respectively [26, 44, 45, 46]. The extent of HSF1 degradation may explain the discrepancies that exist in the presence of UPR activation in different tau transgenic mouse models [3, 26] (Fig 1). Whereas the amount of the mutant human tau in PS19 mice is ~5 times the level of endogenous murine tau, expression of the mutant tau in rTg4510 is approximately 13 fold higher than that of the endogenous tau protein [24, 25]. This higher levels of mutant tau expression in rTg4510 may account for a dramatic decrease of HSF1 protein associated with UPR activation in their brains as compared to PS19.

The consequence of eIF2α phosphorylation appears to be biphasic [47, 48]. Though salubrinal is known to inhibit ER stress via protein synthesis attenuation (to prevent ER protein overload) [49], elevated p-eIF2α by salubrinal rather facilitated UPR-induced HSF1 degradation in N2a-Tau_RD_ ΔK280 of our study (Fig 5). Therefore, furthermore comprehensive consideration of both pathways and their functional outcomes in the cellular context is required. The role of tau aggregates is increasingly recognized in Huntington’s disease (HD). It was previously reported that strong AT8-immunoreactive phosphorylated tau and sarkosyl-insoluble tau were present in human HD patients, exhibiting rod-like tau deposits along neuronal nuclei [50, 51]. Aberrant HSF1 protein degradation in the brains of both HD mice and human HD patients has been recently demonstrated [52]. Therefore, not only in AD but also in HD pathogenesis, tau aggregates might play a critical role in nuclear HSF1 protein degradation, possibly in conjunction with UPR activation as revealed in the current study. Our results are informative of the caution that should be taken in future designs of therapeutic approaches seeking to treat neurodegenerative diseases amenable to UPR inhibition.

## Materials and methods

### Ethics statement

The data on human subjects were analyzed anonymously. All procedures on mice were approved by the Institutional Animal Care and Use Committee (IACUC) at University of Tennessee Health Science Center (UTHSC). Mice were kept in accordance with the institutional guidelines regarding the care and use of laboratory animals.

### Mice and human brain specimens

rTg4510 and rTg21221 mice (maintained in Dr. Hui Zheng’s laboratory, Baylor college of medicine and Dr. Karen H. Ashe’s laboratory, University of Minnesota, respectively) were produced by crossing Tau responder line with CaMKIIa-tTA transactivator line and the mouse brain tissue lysates were used in this study. PS19 mice were obtained on a C57BL/6J genetic background from Jackson laboratory (JAX). HSF1 +/- mice (JAX) were crossed once to C57BL/6J and maintained via intercrossing the F1 HSF1+/- mice. To induce UPR activation, five month-old C57BL6 mice were intraperitoneally administered with thapsigargin (2 mg/kg) dissolved in PBS with 10% DMSO. Tissue specimens of Non-AD and AD patients were obtained from the Human Brain and Spinal Fluid Resource Center, which is sponsored by NIHDS/NIMH, National Multiple Sclerosis Society, Department of Veterans Affairs. Cases derived from short-postmortem interval (PMI) autopsies from the University of Kentucky AD Center (UK-ADC) cohort [53]. Premortem clinical evaluations and pathological assessments were as described previously [54]. Frontal cortical sections correspond to Brodmann Area 9. Tissue used for the biochemical analyses were snap-frozen during the autopsy in liquid nitrogen and then stored at -80 ^o^C. The inclusion/exclusion criteria that were applied: PMI <4 hrs; no evidence of frontotemporal dementia (clinically) or frontotemporal lobar degeneration (pathologically); no cancer in the brain parenchyma; and no large infarctions in the brain, or microinfarcts found within 3 cm of the brain tissue samples. The neuropathological features were assessed using standard neuropathological procedures as described in detail elsewhere [54, 55].

### Synaptosomal membrane preparation and sarkosyl insolubility assay

Mouse hippocampus was homogenated into sucrose-HEPES buffer (4 mM HEPES pH 7.4, 320 mM Sucrose, 2 mM EGTA, containing protease and phosphatase inhibitors). Centrifuge the homogenate at 1,000 g for 10 min at 4 ^o^C to remove the pelleted nuclear fraction. The resulting supernatant from further centrifugation at 15,000 g for 15 min at 4 ^o^C yielded the crude synaptosomal pellet. Resuspend the resulting pellet into sucrose-HEPES buffer. The resulting supernatant from further centrifugation at 15,000 g for 15 min at 4 ^o^C was discarded to remove contaminants. The pellet was then lysed in 4 mM pH 7.4 HEPES and incubated at 4 ^o^C for 30 min. The resulting pellet from further centrifugation at 25,000 g for 30 min contained synaptosomal membrane fraction. Sarkosyl-insoluble tau protein from mouse hippocampus was biochemically isolated as described in Calignon et al [56].

### Thioflavin S staining, immunohistochemistry and imaging

Mouse brain tissues and human brain were fixed in 4% paraformaldehyde, followed by sectioning and then blocking and incubation of primary antibody overnight and then AlexaFluor-conjugated secondary antibody. A double-staining protocol was used to compare Tau46-immunoreactivity to thioflavin S staining of plaques in the mouse brain. Mouse brain tissues were immunostained with Tau46 antibody, followed by Thioflavin S staining. Thioflavin S staining procedure was performed as described in Ly et al [57]. Images were captured under a confocal microscope (Olympus America, Center Valley, PA, USA) and Olympus fluorescent microscope.

### Cell cultures and generation of stable cell lines

N2a cells were cultured in a 1:1 mixture of DMEM and Opti-MEM containing 10% fetal bovine serum (FBS). Cells were transfected with plasmids using Lipofectamine 2000 (Invitrogen). For generation of stably transfected cell lines (N2a-Tau_RD_ΔK280), G418 at 1mg/ml was included in the culture medium. Primary cortical and hippocampal neurons were prepared from E17 rat embryos and maintained in neurobasal medium supplemented with 0.8 mm l-glutamine and B27. Primary neurons cultured for 10–18 days *in vitro* (DIV) were used for the study.

### Reagents and plasmids

Plasmid constructs used in transient transfection include full length tau (pcDNA-WT, R406W) and repeated domain tau constructs (pcDNA-Tau_RD_ WT, Tau_RD_ P301L, Tau_RD_ ΔK280); pcDNA-HSF1 WT, S303A and HSF1 Δ156–226; pcDNA-HSP70a5 (BiP/GRP78). HSF1 WT and HSF1 S303A were generously given by Dr. Dennis Thiele at Duke University. HSF1 Δ156–226 (trimerization mutant) was cloned by using a method of site-directed mutagenesis (Agilent Technologies). Tunicamycin, thapsigargin, salubrinal, resveratrol, piceid, celastrol, rapamycin, and riluzole were all purchased from Sigma. siRNA oligomers for CHOP and BiP were purchased from Sigma.

### Protein analysis by western blots

Tissues and cells were lysed using RIPA buffer (10 mM Tris-Cl (pH 8.0), 1 mM EDTA, 1% Triton X-100, 0.1% sodium deoxycholate, 0.1% SDS, 140 mM NaCl) containing protease inhibitor (leupeptin, pepstatin A, phenylmethylsulfonyl fluoride (PMSF), aproptinin). The resulting lysates were subjected to western blots. Western blots were performed as previously described [19]. Both monoclonal antibody (Santa Cruz, E-4) and polyclonal antibody (Cell signaling, 4356) were used to study HSF1 protein expression. Both antibodies detected ~ 82 kDa HSF1 but showed different banding patterns. p-PERK/p-eIF2α/ eIF2α/BiP/CHOP/Tau (Tau46) (these six from Cell signaling) were also used in the study. Tau (Tau46) mouse monoclonal antibody detects all six isoforms of tau based on the amino acid sequence. For phosphorylated tau, two specific antibodies of AT8 (MN1020) and AT100 (MN1060) were purchased from Thermofisherscientific. Western quantification was based on the intensity of interested signal using densitometry and ImageJ software program.

### MTT (3-(4,5-dimethylthiazol-2-yl)-2,5-diphenyltetrazolium bromide) assay

Cells in a 24 well-plate were incubated with 10 μl of the 12 mM MTT stock solution in a total 100 μl fresh culture medium for 2~3 hrs at 37°C. Reaction was ceased by incubation with 100 μl of the SDS-HCl solution for 2.5 hrs. Absorbance at 570 nm was detected by Beckman Coulter DTX 880 multimode detector.

### Statistical analysis

All statistical analysis was performed by Student’s t-test for two groups’ comparison and one-way ANOVA with a Tukey test for multiple comparisons. Quantitative data in all graphs were presented by means ± SEM (standard error of mean).

## Supporting information

S1 Fig Increase in tau phosphorylation and CHOP induction were not detected in whole brain lysates of 10 month-old HSF1 HSF1 +/- and hippocampus of HSF1+/- at 6 months of age. (A) Immunoreactive band of ~ 60 kDa tau isoform (▲) was detected after long exposure of the blot. *: ~ 68 kDa tau. Arrow heads indicate ~ 64 kDa tau. (B) Western blot was performed on the whole brain lysates of 9 month-old WT and HSF1 +/- to detect protein expression levels of p-Tau (Ser202/Thr205) and CHOP. **(C)** Sarkosyl soluble p-Tau (Ser202/Thr205) from hippocampus of aged HSF1+/- and WT control.
(TIFF)

S2 Fig Double staining of thioflavin S and Tau46 in mouse hippocampus. (A) A representative image captured by fluorescent microscopy showing the absence of thioflavin S-positive aggregates in the hippocampus of wild-type control. (B) Representative confocal images of double staining (Thioflavin S: green, Tau46: red) in the hippocampus of HSF1+/- at 13 months of age. scale bar: 30 μM.
(TIFF)

S3 Fig Chemically induced ER stress in primary neurons. Rapid eIF2a- CHOP activation and tau phosphorylation in response to tunicamycin treatment in rat hippocampal neurons. Rat primary hippocampal neurons were administered with tunicamycin (TM, 1 μM) for short time periods (1–5 hrs). *: ~68 kDa tau. Arrow heads indicate ~64 kDa tau. ▲: ~ 60 kDa tau isoform.
(TIFF)

S4 Fig Measurement of Hsp70 mRNA levels in N2a cells overexpressing different HSF1 constructs and cells treated with celastrol. N2a cells were transfected with either HSF1 WT or HSF1 S303A (constitutively active) or HSF1Δ156–226 (trimerization mutant) or treated with celastrol. Their Hsp70 mRNA expression levels were evaluated by RT-PCR. A graph indicates relative Hsp70 mRNA levels normalized to GAPDH in five groups of cells.
(TIFF)

S5 Fig Tau (full-length) overexpression and Tau RD ΔK280 in N2a cells. (A) Overexpressed full-length Tau WT, Tau P301L, and Tau R406W in N2a cells did not significantly alter HSF1 protein levels. After N2a cells were transfected with full-length mutant tau (P301L, R406W), 48 hrs later, their HSF1 protein expression levels were evaluated by western blot. (B) Highly poly-ubiquitinated HSF1 protein in N2a- Tau_RD_ ΔK280 detected by *in vivo* ubiquitination assay (Detailed methods described in Kim *et al*., 2016). IgG used as a control.
(TIFF)

S6 Fig Protein expression levels of ERAD-associated E3 ubiquitin-protein ligase HRD1 and Sirtuin 1 (SIRT1) in rTg (tauP301L)4510 mice. Whole brain lysates of 7 month-old rTg (tauP301L)4510 mice were subjected to western blot.
(TIFF)
