# Supplementary figures and images for: Bidirectional interplay of HSF1 degradation and UPR activation promotes tau hyperphosphorylation
Source: PLoS Genet. 2017 Jul 5;13(7):e1006849. doi: 10.1371/journal.pgen.1006849 (PMC5517072; doi:10.1371/journal.pgen.1006849)

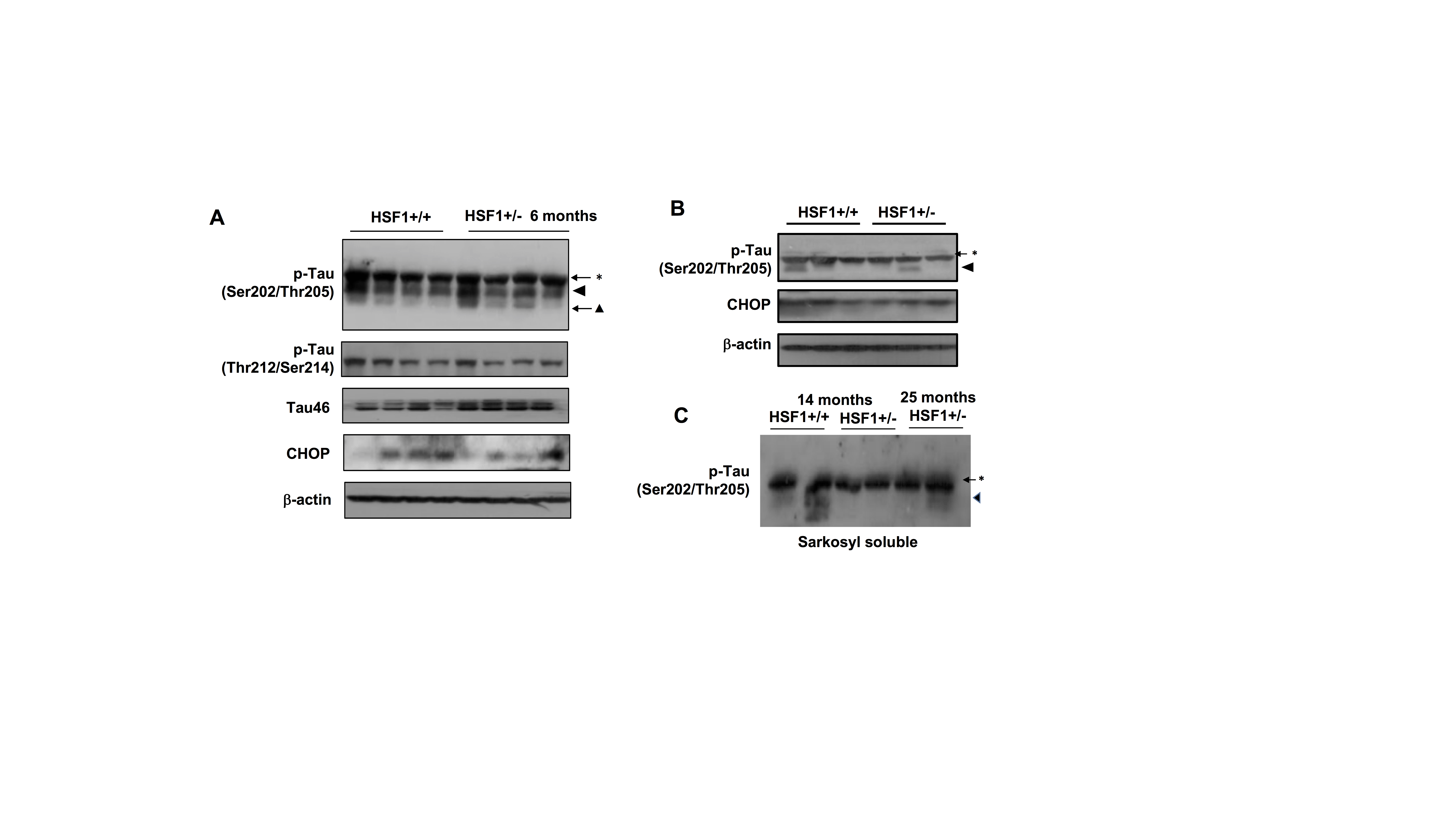

Supplement: S1 Fig — (A) Immunoreactive band of ~ 60 kDa tau isoform (▲) was detected after long exposure of the blot. *: ~ 68 kDa tau. Arrow heads indicate ~ 64 kDa tau. (B) Western blot was performed on the whole brain lysates of 9 month-old WT and HSF1 +/- to detect protein expression levels of p-Tau (Ser202/Thr205) and CHOP. (C) Sarkosyl soluble p-Tau (Ser202/Thr205) from hippocampus of aged HSF1+/- and WT control. (TIFF) [file pgen.1006849.s001.tiff]

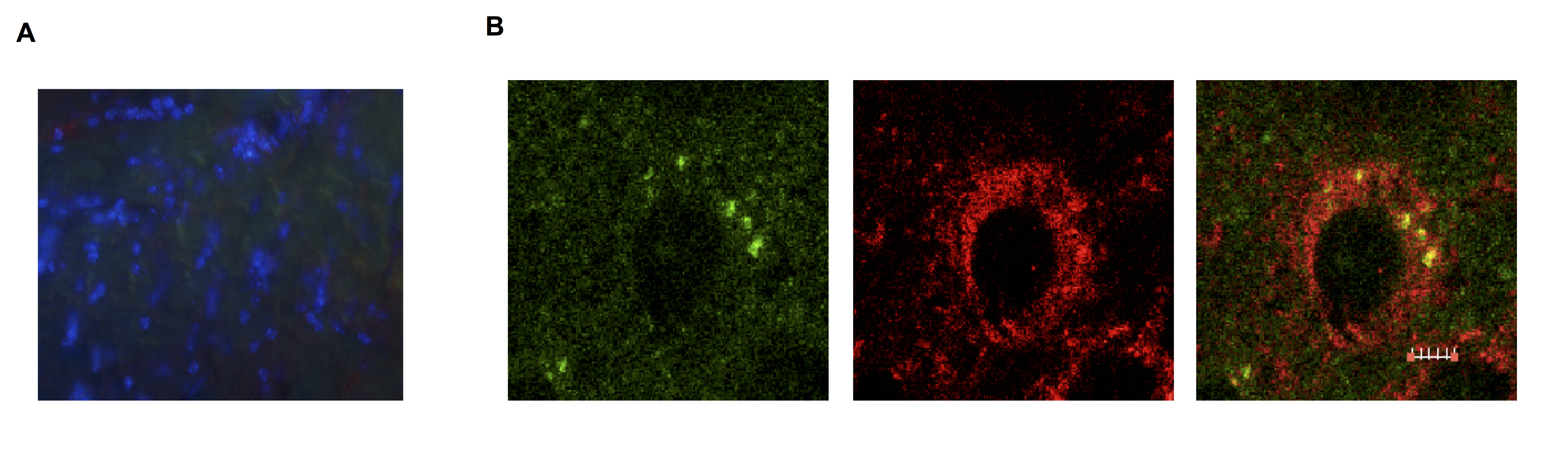

Supplement: S2 Fig — (A) A representative image captured by fluorescent microscopy showing the absence of thioflavin S-positive aggregates in the hippocampus of wild-type control. (B) Representative confocal images of double staining (Thioflavin S: green, Tau46: red) in the hippocampus of HSF1+/- at 13 months of age. scale bar: 30 μM. (TIFF) [file pgen.1006849.s002.tiff]

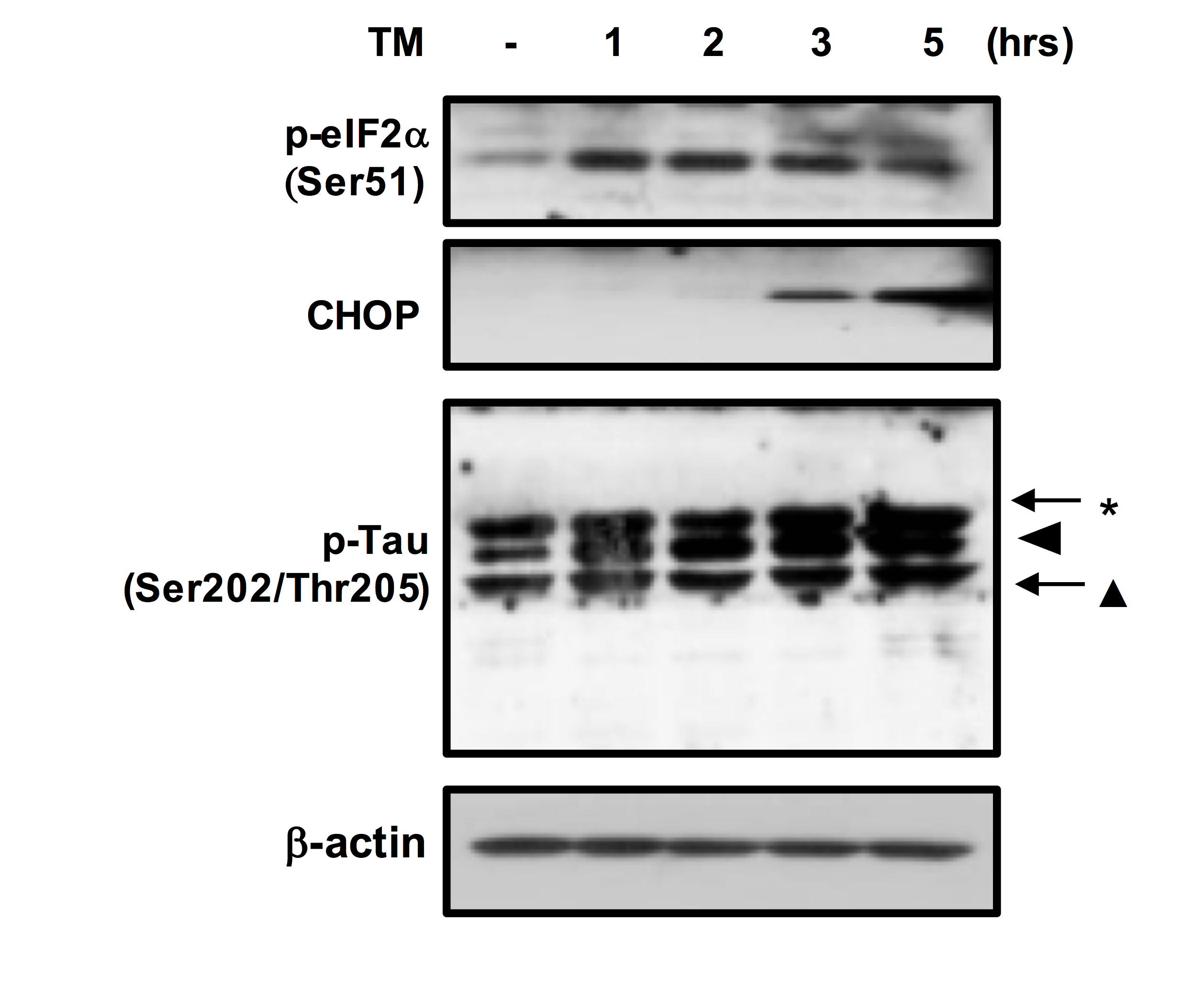

Supplement: S3 Fig — Rapid eIF2a- CHOP activation and tau phosphorylation in response to tunicamycin treatment in rat hippocampal neurons. Rat primary hippocampal neurons were administered with tunicamycin (TM, 1 μM) for short time periods (1–5 hrs). *: ~68 kDa tau. Arrow heads indicate ~64 kDa tau. ▲: ~ 60 kDa tau isoform. (TIFF) [file pgen.1006849.s003.tiff]

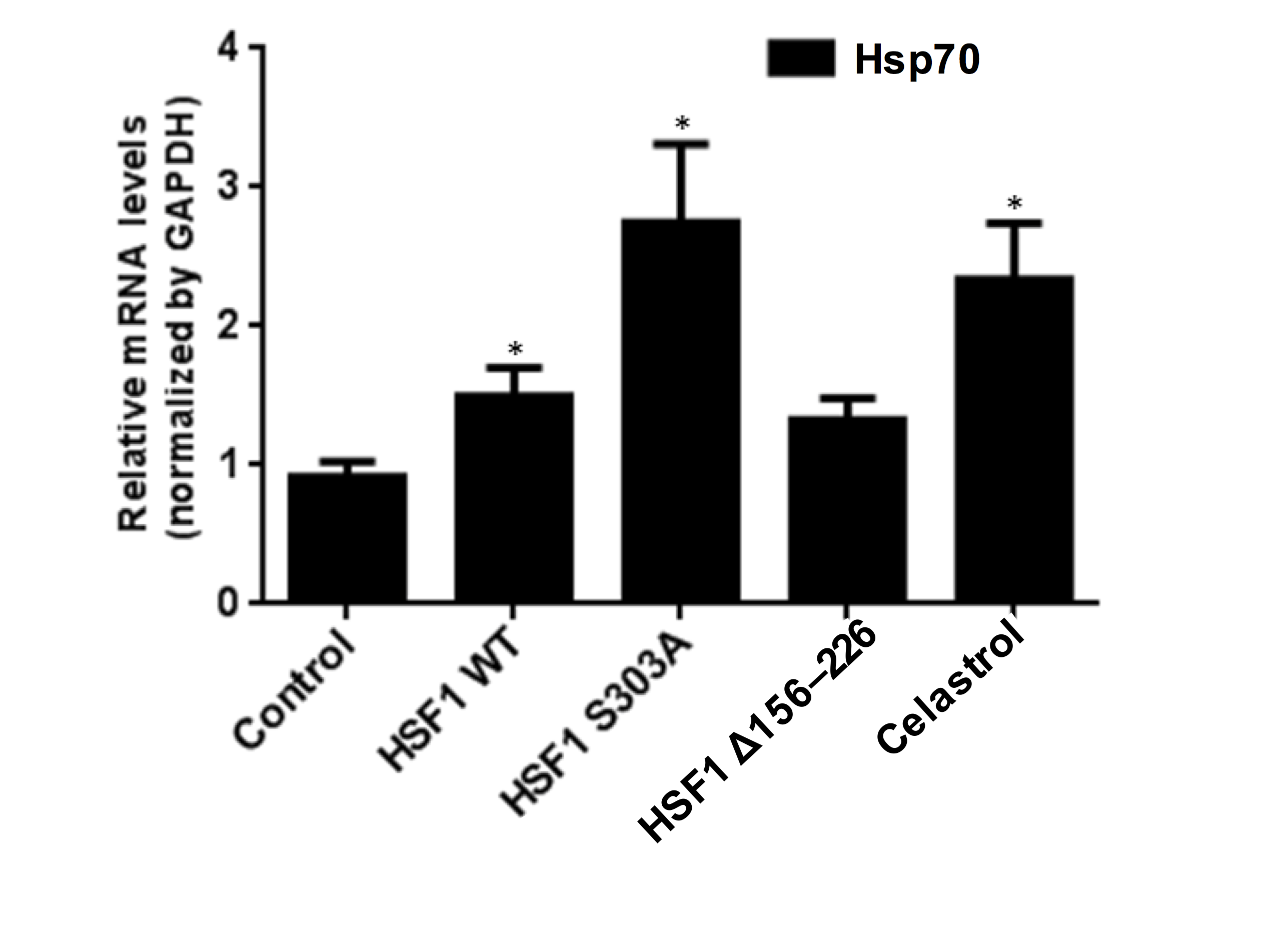

Supplement: S4 Fig — N2a cells were transfected with either HSF1 WT or HSF1 S303A (constitutively active) or HSF1Δ156–226 (trimerization mutant) or treated with celastrol. Their Hsp70 mRNA expression levels were evaluated by RT-PCR. A graph indicates relative Hsp70 mRNA levels normalized to GAPDH in five groups of cells. (TIFF) [file pgen.1006849.s004.tiff]

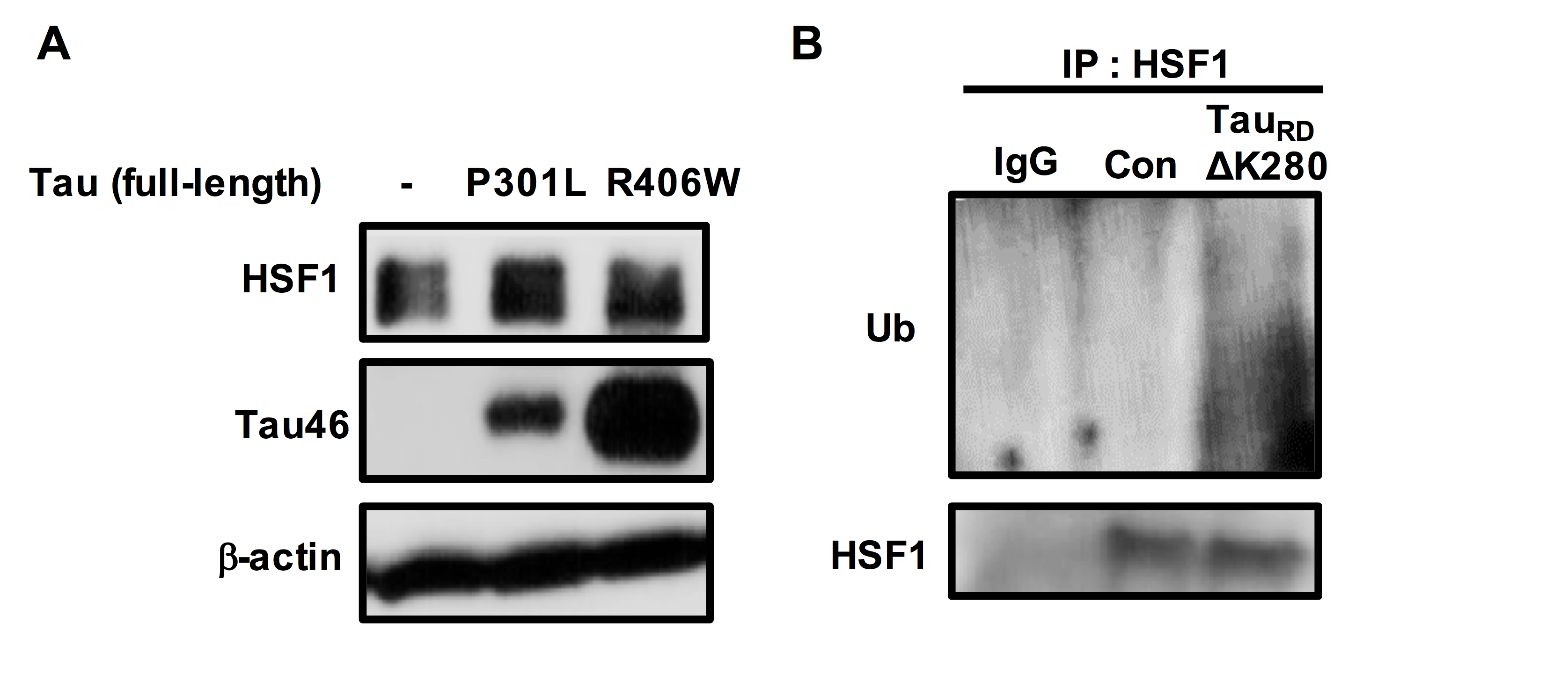

Supplement: S5 Fig — (A) Overexpressed full-length Tau WT, Tau P301L, and Tau R406W in N2a cells did not significantly alter HSF1 protein levels. After N2a cells were transfected with full-length mutant tau (P301L, R406W), 48 hrs later, their HSF1 protein expression levels were evaluated by western blot. (B) Highly poly-ubiquitinated HSF1 protein in N2a- TauRD ΔK280 detected by in vivo ubiquitination assay (Detailed methods described in Kim et al., 2016). IgG used as a control. (TIFF) [file pgen.1006849.s005.tiff]

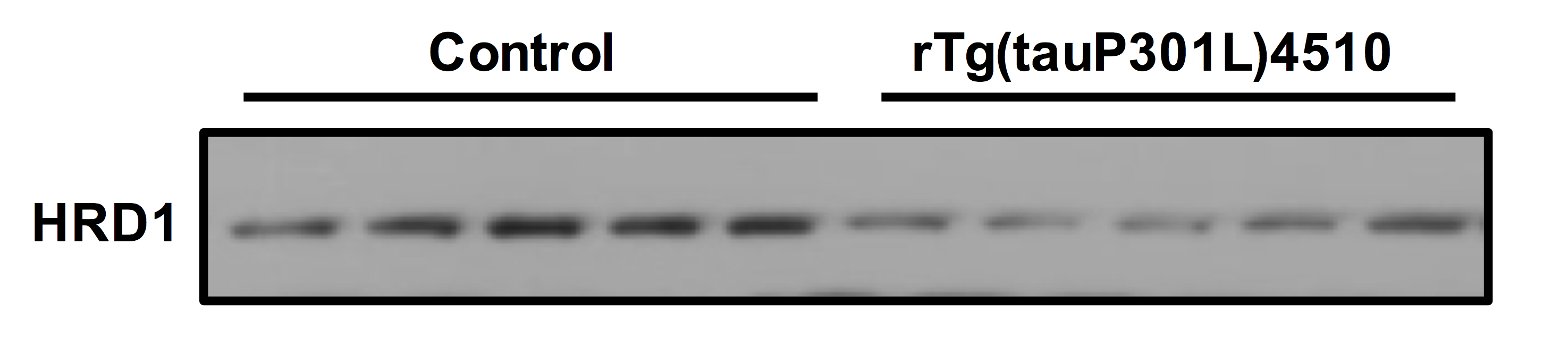

Supplement: S6 Fig — Whole brain lysates of 7 month-old rTg (tauP301L)4510 mice were subjected to western blot. (TIFF) [file pgen.1006849.s006.tiff]
